# Cost of illness of atrial fibrillation: a nationwide study of societal impact

**DOI:** 10.1186/s12913-017-2652-y

**Published:** 2017-11-10

**Authors:** Søren Paaske Johnsen, Lene Worsaae Dalby, Tomas Täckström, Jens Olsen, Anina Fraschke

**Affiliations:** 1 Department of Clinical Epidemiology, Aarhus University Hospital, Olof Palmes Allé 43-45, 8200 Aarhus N, Denmark; 2 Bayer Denmark, Copenhagen, Denmark; 3 Bayer Sweden, Solna, Sweden; 4 Incentive, Holte, Denmark

**Keywords:** Atrial fibrillation, Cost of illness, Costs, Anticoagulants

## Abstract

**Background:**

The prevalence of atrial fibrillation is increasing rapidly; however, to date, population-based data are lacking on the attributable cost of illness of atrial fibrillation from a societal perspective, including both direct and indirect costs.

**Methods:**

The study was an incidence-based cost-of-illness study based on national registries covering the entire population of Denmark. We identified all patients with a first-time hospital diagnosis of atrial fibrillation between 2001 and 2012. For every atrial fibrillation patient, we identified three age- and sex-matched controls from the general population. Both the total and the attributable costs of atrial fibrillation were estimated based on individual level information on hospital care (in- and out-patient contacts), primary sector care, use of prescription drugs and productivity loss.

**Results:**

Average 3-year societal costs per patient attributable to atrial fibrillation were estimated to be €20,403–26,544 during the study period. The costs were highest during the first year after diagnosis of atrial fibrillation. Admission costs constituted the largest cost component, whereas primary sector costs and medicine costs only constituted minor components. The attributable costs were more than two-fold higher among patients experiencing a stroke. The total 3-year cost attributable to atrial fibrillation in Denmark was estimated to be €219–295 million.

**Conclusions:**

The societal costs attributable to atrial fibrillation are significant. Reducing the need for hospitalizations, in particular from stroke, is a key factor in controlling the costs.

## Background

Atrial fibrillation (AF) is the most common cardiac arrhythmia and a major modifiable risk factor for ischaemic stroke. The prevalence of diagnosed AF in the general population was 2.9% in a recent Swedish study, which strongly indicates that the prevalence has been underestimated in previous studies [1, 2]. The true prevalence is even higher due to a substantial number of patients with clinically silent AF [3]. The risk of AF is strongly related to age, and ageing populations, improved care and increased diagnostic awareness has led to a dramatic increase in the prevalence of AF over the past decades. Over 6 million Europeans currently suffer from AF, and projections suggest that the prevalence of AF will more than double by 2050 [4, 5].

AF represents a challenge not only for the individual patient and family, but also constitutes a major economic challenge for healthcare systems and societies as a whole because of the high prevalence and the constrained public budgets. Consequently, there is an increasing need to estimate the costs of AF and determine the distribution of different cost components, as it is becoming increasingly critical to take costs into account in clinical decision making [6–8]. A number of studies on cost of illness of atrial fibrillation have been reported; however, only a few have been nationwide, and up-to-date information on the societal costs attributable to AF are lacking [7, 9–11].

We, therefore, designed a nationwide cost-of-illness study using Danish registries covering the entire population with the aim of estimating the societal costs of AF, including both a total and attributable cost approach.

## Methods

This study was designed as a historical, registry-based cost-of-illness analysis. Healthcare provision in Denmark is publicly funded with equal access and includes no co-payment by the patient in the hospital sector and only a small amount of co-payment for some services in the secondary healthcare sector. Hence, except for some minor co-payment for out-of-hospital pharmacological therapy, all services related to AF (and the complications related to AF care, including bleeding and thromboembolic disease) are free of charge from the patient perspective. In Denmark, there is a long tradition of systematic recording of health service provisions, for example, and it is possible to access and combine a number of high-quality, exhaustive registries at the individual-patient level, with the use of the patient’s encrypted social security number. Danish legislation permits researchers and others to access the databases. Ethics committee approval and written informed consent are not required for registry-based research, according to Danish law.

### Patients with atrial fibrillation and general population controls

New (incident) AF patients diagnosed in the period 2001–2012 (inclusive of both years) were identified in the National Patient Registry (*International Classification of Diseases,* 10th edition [ICD-10] code I48) [12]. The National Patient Registry contains information on all patients discharged from all Danish non-psychiatric hospitals since 1977 and all emergency room and outpatient specialty clinic visits since 1995. Patients with contacts (admissions or outpatient visits) with AF registered as primary or secondary diagnosis were defined as AF patients. In order to identify only new cases of AF, a washout period of 5 years was used, implying that individuals who have been in contact with a hospital and diagnosed with AF in the period 1996–2000 were excluded from the AF population.

A gender- and age-matched control group of individuals free of AF was identified using the Danish population registry (the Danish Civil Registry) [13]. For each AF patient, three controls were identified. The same individual was allowed to be used as a control multiple times in different years, but not in the same year. Furthermore, controls for AF patients were only between 18 and 89 years old at incidence as there was lack of potential controls for older AF patients (aged ≥90 years). Therefore, the cost analyses, which depend on the matching, only include 18- to 89-year-old patients.

All included patients had at least 1 year of history and follow-up included in the registries (i.e. a patient diagnosed in November 2012 was ‘followed’ in the registries until November 2013).

### Costs of AF

A societal perspective was applied including the following six cost types:

- Primary sector costs
- Outpatient costs
- Hospital admission costs
- Medicine costs
- Home care costs
- Productivity loss (productivity costs)

Primary sector costs were obtained from the National Health Service Registry, which holds data on all contacts in the primary care sector, including contacts with general practitioners and private practice specialists [14]. The fee paid to the healthcare professional (e.g. general practitioner) was applied as unit cost estimate for services provided in the primary care sector.

Data on hospital costs were obtained from the National Patient Registry. We used the Danish outpatient charges (DAGS charges) as unit cost estimate for each outpatient contact (including emergency room visits) and the Diagnosis Related Groups (DRG) charges for admissions.

Data on medicine costs were obtained from the Registry of Medicinal Product Statistics, which holds information on all acquisitions of prescription medicine at Danish pharmacies, including prices [15]. The market price was applied as unit cost estimate for each acquisition.

Home care costs included both costs at own home and from nursing homes. The Danish municipalities provide these home care services, and the data originate from the municipalities’ own registry, which was accessed via Statistics Denmark. The data were available from 2008 onwards. Data on care in nursing homes were, however, only available until 2012. The registry includes information about the services (measured in minutes) each individual has been granted every month with respect to both nursing and practical help. In order to approximate this into a cost estimate, gross wages for nurses and home-helpers were applied from the Salary Data Office of Municipalities and Regions. In order to estimate an hourly salary, the annual salary was divided by 1628 h, which is the number of effective working hours per year given a 37-h working week. To translate the wage into a cost, a 75% overhead charge was added covering administration costs and transportation.

Productivity losses were estimated using weekly employment data from the DREAM database (The longitudinal database of The Danish Agency for Labour Market and Recruitment). The DREAM database includes all individuals who have received labour market-related transfer payments since 1991 [16]. With DREAM, it was estimated what fraction of the baseline year as well as the 1st, 2nd, and 3rd year after AF an individual was employed. Individuals who have never received public transfers do not appear in DREAM. Individuals, who do not appear in DREAM and who are aged ≤65 years, were treated as individuals who have worked for the entire year. Furthermore, individuals aged >65 years were treated as if they were not employed (i.e. retired) independent of their employment status according to DREAM. This was done in order to create consistency in the way individuals who appear in DREAM and those who do not were treated.

The productivity value for each individual was estimated by multiplying the estimated yearly employment fraction with a gender-specific gross average wage, adjusted for the number of effective working hours per week performed by each gender. With the productivity value of each individual, the productivity loss in year t was estimated as the productivity value in year t minus the productivity value in the baseline year. This was defined as the total productivity loss (productivity costs). The attributable cost in terms of productivity loss was the productivity loss for the AF patient minus the productivity loss for the controls. All costs were inflated to 2013 price levels. Primary sector costs, outpatient costs and hospital admission costs were inflated using the price and salary index from the Danish Regions. Similarly, medicine costs were inflated using the medicine price index from Danish Regions. For the estimates of home care costs and productivity losses, constant 2013 prices were used for salaries and earnings, respectively. The salaries for nurses and home-helpers were identified using yearly gross wages for municipally employed nurses and home-helpers, whereas the productivity values were estimated using per-hour earnings from the private sector. Future costs in year 2 and in year 3 were discounted by 4% per year.

### Data analysis

We first determined the yearly incidence and prevalence of AF. The AF prevalence estimates were based on the AF patients diagnosed between 2001 and 2012. The number of prevalent AF patients in a given year (by the end of the year) was, thus, the number of new cases plus the number of AF patients from previous years minus the number of deaths. The denominator was the total Danish population aged 18 years or older in the given year. The mortality among AF patients relative to the controls was measured as the percentage of both AF patients and controls who died in the 1st, 2nd and 3rd year after AF diagnosis. Information about deaths was retrieved from the National Causes of Death Registry. Information on the general population, including age- and sex-distribution, was obtained from Statistics Denmark.

We then estimated the costs of AF, including the costs in the 1st, 2nd and 3rd year after the AF diagnosis, using both a total cost and attributable cost approach.

The total costs for every individual *i* in year t was defined as:

$$ Total\ {cost}_{i,t}^{AF}={\sum}_{x=1}^X{Cost}_{i,x,t}^{AF}-{Cost}_{i,x,0}^{AF}, $$

where AF is an abbreviation for “AF patient”, t is the year of interest (i.e. 1st, 2nd or 3rd year after AF diagnosis), 0 is the year leading up to the AF diagnosis, x is the type of cost (e.g. outpatient cost, admission cost, etc.). If an AF patient was diagnosed on 1 April 2008, the total costs in the 1st year after AF would be his or her costs from 1 April 2008 to 31 March 2009 minus his or her costs from 1 April 2007 to 31 March 2008.

The attributable costs are the total costs of AF minus the total costs among the controls in accordance with previous publications [17, 18]. Hence, attributable costs were estimated as a difference-in-difference in which the difference between costs for cases (AF patients) and controls was regarded as being attributable to the AF.

$$ Attributable\ {cost}_t^{AF}=\sum \limits_{x=1}^X\left({Cost}_{x,t}^{AF}-{Cost}_{x,0}^{AF}\right)-\left({Cost}_{x,t}^C-{Cost}_{x,0}^C\right) $$

Since AF patients and controls were not necessarily part of the analysis during the entire 3 years, due to either death or the conclusion of the study, their costs were weighted by the fraction of the time they were part of the analysis.

Finally, we performed a subgroup analysis among AF patients subsequently diagnosed with ischaemic stroke (ICD-10 codes I63, I64). This was done in order to assess the cost impact of ischaemic stroke in AF patients. That is, in the National Patient Registry for the 1st, 2nd or 3rd year following the AF diagnosis, we identified all AF patients, with a subsequent hospital contact for ischaemic or non-specified stroke. In order to ensure that only new cases of stroke were included, a 5-year wash out period was applied.

The total cost of AF and subsequent stroke was, for every patient, a measure of his or her costs in year t (after the AF diagnosis) minus his or her costs in the baseline year (i.e. the year up to the AF diagnosis). In this subanalysis, patients were also categorized according to the incidence year of stroke (i.e. AF patients, stroke year 1; AF patients, stroke year 2; and AF patients, stroke year 3 – where stroke year 1 indicates that the patient were diagnosed with stroke within the first year following AF).

The attributable cost was estimated as the total cost of an AF patient with subsequent stroke minus the total cost among AF patients without stroke (i.e. AF patients without stroke are the controls). Again, attributable costs were estimated as a difference-in-difference.

## Results

A total of 193,265 patients were registered with a first-time hospital diagnosis of AF during the study period from 2001 to 2012. The yearly incidence varied from 15,066 to 18,094, which corresponded to incidence rates of 3.6–4.1 per 1000 person-years (Table 1). The incidence and overall incidence rate was highest in the most recent years; however, the age-specific incidence rates in general appeared stable throughout the study period (Table 1).

**Table 1:** Incidence rate (per 1000 person-years) of first-time hospital contacts for atrial fibrillation in Denmark 2001–2012

		2001	2002	2003	2004	2005	2006	2007	2008	2009	2010	22,011	2012
Women	18–65 y	0.7	0.7	0.7	0.7	0.7	0.8	0.8	0.8	0.9	0.8	0.9	0.9
	66–75 y	7.7	7.8	7.2	7.1	7.0	7.1	7.4	7.3	7.1	7.6	7.8	7.6
	76+ y	20.4	20.6	20.9	20.0	20.2	20.1	20.2	19.9	20.7	21.5	22.2	21.8
Men	18–65 y	1.5	1.4	1.5	1.5	1.6	1.6	1.6	1.7	1.7	1.7	1.8	1.7
	66–75 y	12.4	12.0	11.8	11.5	11.5	11.1	11.4	11.3	11.3	12.0	12.3	11.9
	76+ y	26.3	26.3	25.4	25.8	24.7	24.0	24.3	23.9	23.9	25.5	25.5	26.8
Total		3.7	3.7	3.6	3.6	3.7	3.6	3.7	3.7	3.8	4.0	4.1	4.1

The prevalence of AF in the Danish population in 2012 (i.e. the latest available year) was 107,526, corresponding to a prevalence proportion of 24.6 per 1000 persons (Table 2). The highest prevalence proportion was observed among persons aged ≥76 years, particularly men.

**Table 2:** Prevalence and prevalence proportion (per 1000 persons) of patients with a hospital diagnosis of atrial fibrillation in Denmark in 2012

		Prevalence	Prevalence proportion
Women	18–65 y	13,686	7.9
	66–75 y	13,860	50.2
	76+ y	19,457	89.2
Men	18–65 y	28,093	16.0
	66–75 y	18,794	73.4
	76+ y	13,636	96.8
Total		107,526	24.6

### Individual-level cost of AF

**Fig. 1:**
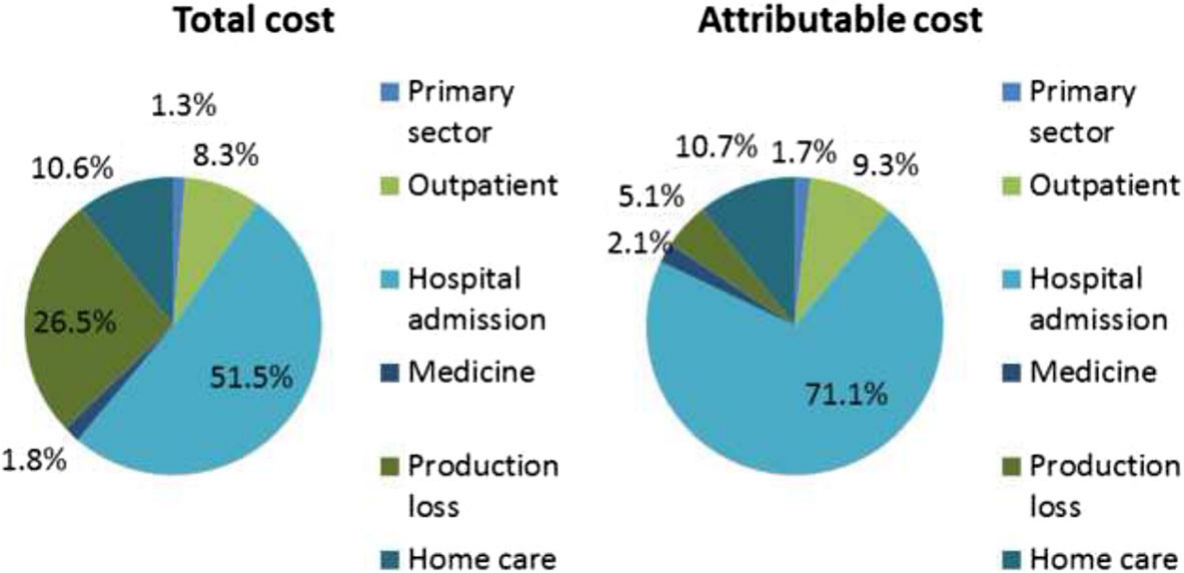
Distribution of total* and attributable** average individual costs in year 1-3 after a first-time hospital diagnosis of atrial fibrillation. Data are from the latest available year with up to 3 years of follow-up (i.e., patients diagnosed with atrial fibrillation in 2010). *Total costs after AF diagnosis (including primary sector, outpatient, hospital admission, medicine, production loss and home care) minus the costs in the preceding year. **Total costs of AF minus the total costs among the controls

The total and attributable average individual-level cost of AF for years 1–3 after the first AF diagnosis are presented in Table 3. That is, the average cost per AF patient during the 1st, 2nd and 3rd years after the AF diagnosis date minus the average cost per patient the year before the AF diagnosis date. The table includes data on patients diagnosed in 2010, which was the latest available year enabling 3 years of follow-up. The analyses were, however, also done for the preceding years and the results were comparable to the population from 2010 (data not shown). Using the total cost approach, the total cost per AF patient, 1–3 year after the AF incidence date, was estimated to be €32,156 for patients diagnosed in 2010. The amount varied from €27,516 to €36,701 during the study period. The costs were highest during the first year. Admission costs constitute the relative highest cost component followed by cost of lost productivity (Fig. 1). Primary sector and medicine costs constituted only small proportions of the total costs (i.e. less than 2% each).

**Table 3:** Total^a^ and attributable^b^ average cost per individual in years 1–3 after a first-time hospital diagnosis of atrial fibrillation

	Cost, year 1 (*N* = 15,237 AF patients)		Cost, year 2 (*N* = 11,403 AF patients)		Cost, year 3 (*N* = 9734 AF patients)		Sum, year 1–3	
	Total	Attributable	Total	Attributable	Total	Attributable	Total	Attributable
Primary sector costs	222	206	121	103	75	80	418	389
Outpatient costs	1708	1533	567	388	399	203	2673	2125
Hospital admission costs	6657	6336	5457	5398	4456	4531	16,570	16,265
Medicine costs	169	143	186	155	210	186	564	484
Productivity loss	2012	824	2697	191	3824	160	8533	1176
Home care costs	1237	992	1241	819	920	629	3397	2439
Total	12,005	10,034	10,268	7055	9883	5788	32,156	22,878

^a^Total costs after AF diagnosis was defined as the average cost per AF-patient 1st, 2nd and 3rd year after the AF diagnosis date (including primary sector, outpatient, hospital admission, medicine, productivity loss and home care) minus the average cost per patient the year before the AF diagnosis date

^b^The total costs of AF minus the total costs among the controls. Data are from the latest available year with up to 3 years of follow-up (i.e. patients diagnosed with atrial fibrillation in 2010). All costs are in euros and based on 2013 prices

When using the attributable cost approach, the average cost per AF patient, 1–3 years after the AF incidence date, was €22,878 for patients diagnosed in 2010 (Table 3). During the study period, this amount varied from €20,403 to €26,544. The costs were also highest within the first year after AF diagnosis, and admission costs constituted the largest cost component (71.1% for patients diagnosed in 2010) (Fig. 1).

In Table 4, the estimated total cost per AF patient, with or without subsequent ischaemic stroke, is shown. As for Table 3, only data on patients diagnosed with AF in 2010 are shown; however, similar findings were seen for patients diagnosed in the preceding years. The costs over a 3-year period for AF patients experiencing stroke within the first year after AF diagnosis were markedly higher than the costs for AF patients free of stroke. The primary cost driver was the admission costs; however, home care costs for AF patients with subsequent stroke are also high compared with the similar costs for AF patients free of stroke. By contrast, the costs for outpatient care were marginally lower for patients with ischaemic stroke. For both AF patients free of stroke and AF patients with subsequent stroke, the medicine costs are relatively low, but these costs are highest for AF patients with stroke.

**Table 4:** Total average cost per individual in years 1–3 after a first-time hospital diagnosis of atrial fibrillation according to occurrence of ischaemic stroke within the first year after diagnosis

	Patients without ischemic stroke Sum of costs, years 1–3 (*N* = 13,492 AF patients)	Patients with ischemic stroke Sum of costs, years 1–3 (*N* = 682 AF patients)	Average costs attributable to ischemic stroke Sum of costs, years 1–3
Primary sector costs	365	1412	1047
Outpatient costs	2670	2314	−357
Hospital admission costs	15,112	63,692	48,580
Medicine costs	554	861	307
Productivity loss	8660	8919	258
Home care costs	2703	12,312	9609
Total	30,066	89,510	59,443

Attributable cost reflects cost attributable to ischaemic stroke and was estimated as the total cost of AF patient with subsequent stroke minus the total cost among AF patients without stroke. Data are from the latest available year with up to 3 years of follow-up (i.e. patients diagnosed with atrial fibrillation in 2010). All costs are in euros and based on 2013 prices

### National-level cost of AF

Table 5 presents estimates for the total and attributable cost for AF patients in Denmark (given the total cost/attributable cost per patient approach and the number of patients, N). For the yearly cohorts of incident AF patients, the total cost in Denmark during the first 3 years varied from €297 million to €428 million during the study period. For patients diagnosed in 2010, the total costs were €396,204,346.

**Table 5:** Total and attributable cost at national level in years 1–3 after a first-time hospital diagnosis of atrial fibrillation

	Cost, year 1 (N = 15,237 AF patients)		Cost, year 2 (N = 11,403 AF patients)		Cost, year 3 (N = 9734 AF patients)		Sum, years 1–3	
	Total*	Attributable**	Total*	Attributable**	Total*	Attributable**	Total*	Attributable**
Primary sector costs	3,380,355	3,131,643	1,379,417	1,179,182	732,007	774,842	5,491,779	5,085,667
Outpatient costs	26,019,512	23,363,782	6,460,893	4,425,880	3,883,182	1,979,153	36,363,587	29,768,816
Hospital admission costs	101,439,528	96,538,218	62,222,657	61,554,643	43,377,643	44,102,582	207,039,828	202,195,443
Medicine costs	2,569,889	2,183,094	2,116,885	1,771,149	2,041,190	1,807,702	6,727,964	5,761,946
Productivity loss	30,659,407	12,562,422	30,754,554	2,183,733	37,219,783	1,556,676	98,633,744	16,302,831
Home care costs	18,848,653	15,113,054	14,148,496	9,336,404	8,950,296	6,120,822	41,947,445	30,570,279
Total	182,917,343	152,892,214	117,082,902	80,450,991	96,204,100	56,341,777	396,204,346	289,684,981

*Total costs after AF diagnosis (including primary sector, outpatient, hospital admission, medicine, productivity loss and home care) minus the costs in the preceding year

**The total costs of AF minus the total costs among the controls

Data are from the latest available year with up to 3 years of follow-up (i.e. patients diagnosed with atrial fibrillation in 2010). All costs are in euros and based on 2013 prices

The corresponding cost attributable to AF in Denmark was estimated to be €219–295 million. Hospital admission costs constituted the majority. Out of the €219–295 million, the direct healthcare cost (primary sector costs, outpatient costs, hospital admission costs and medicine costs) comprised €194–263 million. For patients diagnosed in 2010, the attributable costs were €289,684,981.

## Discussion

AF is characterized by a high incidence and prevalence, particularly among the elderly, as demonstrated in our nationwide study. The societal costs for handling AF and its consequences for the individual patient are substantial, particularly the high costs of hospital admissions in relation to AF, whereas other cost components including cost for primary care and medicine only play a minor role. Prevention of stroke is a key component as illustrated by the huge difference in costs for AF patients with and without a subsequent stroke. Due to the high incidence and prevalence, the costs of AF at a national level were very high.

Our findings confirm and extend previous cost-of-illness studies [7, 9–11]. Our study is, to the best of our knowledge, the first nationwide study aiming to estimate the societal costs attributable to AF. In doing so, we considered both the costs in the year preceding the AF diagnosis and the costs experienced by general population controls without AF. Furthermore, we applied a broad perspective including not only the cost of hospital care (including admissions and outpatient visits), primary sector care and medicine, but also the cost of home care and cost of lost productivity.

The total healthcare costs in Denmark are approximately €14 billion per year. According to our estimates, the total healthcare costs attributable to AF amounted to €194–263 million during our study period, corresponding to 1.3–1.7% of the total healthcare costs. This estimate is in accordance, but most likely more accurate, than a previous UK study, which, based on data from 1995, estimated that the direct costs of AF constituted 0.9–2.4% of the UK healthcare budget in 2000 [11]. The disturbingly high costs are already a challenge to the healthcare systems and appear to correspond or exceed the estimated costs of other major diseases including depression, osteoporosis and breast cancer [7]. The observed growth in the incidence of AF in our study and projections for the future indicate that the impact is likely to grow even further in the coming decades [4].

Looking at the distribution of cost components, it is somewhat difficult to make direct comparisons between the existing cost-of-illness studies due to differences in methodology; however, it is a strikingly consistent finding that hospital admission for AF and AF-related complications is the dominant cost component [6–8]. Stroke is the most feared complication of AF from a patient and clinician perspective, but our findings also emphasize the importance of ensuring effective stroke prevention in AF patients from a cost perspective, as the costs attributable to AF were substantially higher among AF patients who experienced a subsequent stroke. Although the cost implications of stroke in patients with AF are dramatic, they are not unexpected as AF is associated with a higher risk of adverse outcomes following stroke (including increased risk of in-hospital medical complications, longer length of stay, lower functional level, increased case-fatality and, possibly, higher risk of recurrent stroke episodes) [19, 20]. Please note that our findings reflect not only that there are overall differences in the costs between the AF patients with and without ischaemic stroke, but also that there are differences in the distribution of the costs. Thus, costs related to hospital admission were substantially higher among AF patients suffering an ischaemic stroke, whereas the costs for outpatient care were marginally lower. These differences are not unexpected and are in line with clinical experience (e.g. an AF patient without a stroke may initiate oral anticoagulation therapy in an outpatient clinic, whereas an AF patient with a stroke may initiate oral anticoagulation during a stroke-related hospital admission).

The strengths of our study included the nationwide, population-based, controlled study design with inclusion of three matched general population controls for each AF patient, as well as the use of prospectively collected registry data. There is a long tradition for registry-based research in Denmark and the included registries are, in general, considered to be highly accurate as they are all used for administrative purposes. The predictive values of the AF and stroke diagnoses recorded in the National Patient Registry have been examined previously and reported to be more than 90% [21, 22].

The limitations include the use of standard charges and fees as unit costs for admissions, outpatient contacts and GP visits. These may not reflect the true resource use accurately; however, they were considered to be the best available proxies for the true resource use. Furthermore, even though our study applies a broad perspective, including the cost of home care and cost of lost productivity, all costs (e.g. costs for short-term sick leave) were not included. The applied registry (the DREAM database) only included information on long-term sick leave/absence. Other costs for patients and relatives (e.g. transportation time associated with hospital contacts or costs for non-prescription medicine) and possible costs of informal care provided by spouse or another close relative were also not included. Our estimates for the costs of AF are, therefore, likely to be conservative.

## Conclusion

Our study documents the substantial societal costs attributable to AF using nationwide Danish registries covering the entire population. The costs represents a huge challenge for societies in the coming decades due to increasing incidence of AF. Healthcare costs, particularly hospital admissions, are the main cost component and these costs are strongly influenced by the occurrence of stroke, the most important medical complication of AF. Effective prevention of stroke in patients with AF is therefore paramount both from a clinical and economical perspective.

## Abbreviations

AF: atrial fibrillation
DAGS: Danish outpatient charges
DREAM database: The longitudinal database of The Danish Agency for Labour Market and Recruitment
DRG: Diagnosis -Related Groups
ICD-10: International Classification of Diseases, 10th edition
UK: United Kingdom
